# A High‐Riding Patella Is a Feature of Knee Joint Motion During Gait After ACL Reconstruction

**DOI:** 10.1002/jor.26062

**Published:** 2025-03-19

**Authors:** Marcus G. Pandy, Hans A. Gray, Padma N. Ganapam, Adam G. Culvenor, Kay M. Crossley, Shanyuanye Guan

**Affiliations:** ^1^ Department of Mechanical Engineering University of Melbourne Melbourne Victoria Australia; ^2^ Department of Biomedical Engineering University of Melbourne Melbourne Victoria Australia; ^3^ La Trobe Sport and Exercise Medicine Research Centre, School of Allied Health, Human Services and Sport La Trobe University Melbourne Victoria Australia

**Keywords:** bone shape, patella alta, patellar tendon, patellofemoral, tibiofemoral

## Abstract

Accurate measurements of knee joint motion during gait after anterior cruciate ligament reconstruction (ACLR), especially for the patellofemoral joint, are sparse. The aim of this study was to measure six‐degree‐of‐freedom (6‐DOF) patellofemoral and tibiofemoral motion in ACLR and uninjured contralateral knees during gait, and to compare these results to healthy (control) knees. Biplane fluoroscopy was used to measure 6‐DOF patellofemoral and tibiofemoral motion in 15 ACLR participants (26.3 ± 3.9 years) for complete cycles of level walking and downhill walking, and the results were compared to data for 10 healthy individuals (29.8 ± 6.1 years). Mean patellar superior translation, anterior translation, and flexion over the gait cycle were respectively 4.4–5.6 mm greater, 5.4–6.3 mm greater, and 3.7°–7.0° less in the ACLR and contralateral knees compared to controls across both activities (*p* < 0.021). Articular contact was 7.6 mm higher on the femoral trochlea in the ACLR and contralateral knees compared to controls. The patellar tendon was 8.9 mm longer (*p* < 0.001) in the ACLR and contralateral knees compared to controls. Among ACLR participants, 14 out of 30 knees (47%) had an Insall–Salvati ratio ≥ 1.20, indicating patella alta. Mean tibial external rotation and anterior translation over the gait cycle were respectively 3.4°–3.8° greater and 2.6–3.0 mm greater in the ACLR knee compared to controls across both activities (*p* < 0.025). A high‐riding patella in both knees of the ACLR participants was due to a longer patellar tendon. A change in the load‐bearing areas of the femoral trochlea may contribute to the high rate of patellofemoral osteoarthritis seen after ACLR.

## 1 Introduction

Anterior cruciate ligament reconstruction (ACLR) does not reduce the risk of premature knee osteoarthritis (OA) after ACL injury. Radiographic studies indicate that 50% of individuals < 40 years of age develop knee OA 8–12 years after ACL injury, irrespective of conservative or surgical management [1]. More recent magnetic resonance imaging studies suggest that knee cartilage degeneration may occur as early as 6 months after ACLR [2]. While the etiology of knee OA after ACLR remains unclear, altered knee joint motion and loading are thought to contribute to the development and progression of knee OA [3, 4].

Video motion capture of skin‐mounted markers is frequently employed to measure 3D tibiofemoral joint motion during level walking after ACLR [5, 6, 7, 8]. Georgoulis et al. [5] reported an increase in internal tibial rotation in the ACLR knee compared to the healthy (control) knee, whereas Webster and Feller [7] found that the ACLR knee was externally rotated relative to the healthy knee. Scanlan et al. [6] and Webster and Feller [7] found that the ACLR knee was externally rotated relative to the uninjured contralateral knee. Erhart‐Hledik et al. [8] observed an increase in external tibial rotation in the ACLR knee relative to the contralateral knee 2 years after surgery, however the ACLR and contralateral knees were both internally rotated relative to the healthy knee for most of the stance phase. A known limitation of video motion capture is the error incurred by skin marker movement, which may explain some of these conflicting results. Root‐mean‐squared (RMS) errors up to 29 mm for translations and 24° for rotations have been reported for measurements of knee joint motion obtained from video motion capture [9], whereas the femur, tibia, and patella translate and rotate by < 10 mm and < 10° respectively in the transverse and frontal planes during gait [10].

Few studies have recorded tibiofemoral joint motion after ACLR using X‐ray fluoroscopy. Papannagari et al. [11] measured six‐degree‐of‐freedom (6‐DOF) tibiofemoral kinematics (three rotations and three translations) during a static forward lunge and found greater anterior tibial translation and external tibial rotation in the ACLR knee relative to the contralateral knee. Tashman et al. [12] measured 6‐DOF tibiofemoral joint motion after ACLR for part of the stance phase of downhill running at 2.5 m/s. They found no differences in anterior tibial translation between the ACLR knee and contralateral knee; however, the ACLR knee was more externally rotated and more adducted relative to the contralateral knee.

Less is known about changes in patellofemoral joint motion after ACLR despite patellofemoral OA being common and most burdensome after ACLR [13]. Van de Velde et al. [14] used biplane fluoroscopy to measure 6‐DOF patellofemoral kinematics during a quasi‐static forward lunge and found that patellar flexion decreased while patellar shift, rotation, and tilt all increased in the ACLR knee relative to the contralateral knee. They also noted a proximal shift in the location of patellofemoral joint contact in the ACLR knee compared to the contralateral knee. No study to our knowledge has recorded patellofemoral joint motion during walking after ACLR.

The aim of this study was to measure 6‐DOF patellofemoral and tibiofemoral joint motion in the ACLR knee and the contralateral knee for complete cycles of level walking and downhill walking using mobile biplane X‐ray fluoroscopy [15], and to compare these results to those obtained previously for healthy knees from uninjured individuals [16]. Downhill walking was selected because it presents a greater challenge to knee joint stability than level walking. We hypothesized that: (1) There are no significant differences in patellofemoral kinematics between the ACLR, contralateral, and healthy knees in both activities; and (2) External tibial rotation is significantly greater in the ACLR knee compared to the contralateral and healthy knees in both activities.

## 2 Materials and Methods

### 2.1 Participants

Fifteen young adults (8 [53%] female; age: 26.3 ± 3.9 years; height: 174.2 ± 11.6 cm; weight: 76.5 ± 8.4 kg; BMI: 25.3 ± 2.5 kg/m^2^) who had undergone primary unilateral hamstring‐tendon (4‐stranded semitendinosus/gracilis) autograft ACLR 6–15 months before testing were recruited. Exclusion criteria included: (1) < 18 and > 35 years old; (2) < 6 and > 15 months after ACLR; (3) the presence of another condition affecting daily function; and (4) > 50% of the meniscus removed during ACLR. Ten healthy young adults (four [40%] female; age: 29.8 ± 6.1 years; height: 168.0 ± 9.9 cm; weight: 68.3 ± 9.0 kg; BMI: 24.2 ± 2.4 kg/m^2^) with no knee pain or history of lower‐limb injury or surgery were tested in a previous study [16] and served as the control group. The ACLR participants were physically active and participated in competitive sports at least once per week, while those in the control group were predominantly sedentary. Tegner activity level scores for the ACLR group ranged from 4 to 9 before ACL injury and were not available for the controls. Ethics approval for the study was granted by the University of Melbourne (Project ID 12358) and all participants provided informed consent.

### 2.2 Computed Tomography (CT) Imaging

CT scans (Brilliance 64, Philips; slice thickness = 1.0 mm; voxel size = 0.46 × 0.46 × 0.50 mm^3^) of the ACLR and uninjured contralateral knees were acquired with the participant lying supine and each knee extended. CT scans of the right knee of each participant in the control group were acquired previously [17]. The CT scans were segmented using 3D Slicer [18], and volumetric models of the distal femur, proximal tibia, and patella were created for each knee.

### 2.3 Gait Experiments

Each ACLR participant wore a lead vest, short pants, and sandals. Forty‐five reflective markers were attached to the skin on predetermined landmarks identified on the torso and all four extremities [19]. The participant walked on level ground and on a 10° downward slope (level walking and downhill walking, respectively) at their self‐selected speed, while full‐body motion, ground reaction forces (GRFs), and biplane X‐ray images were recorded simultaneously. The experimental protocol was identical to that described previously for the healthy participants [16], except that, for each ACLR participant, biplane X‐ray images of the ACLR knee and uninjured contralateral knee were recorded in two separate trials for each activity.

Full‐body motion was measured using an 8‐camera video motion capture system (VICON, Oxford Metrics, UK) sampling at 200 Hz. GRFs were recorded using three force plates (AMTI, Watertown, MA, USA) sampling at 1000 Hz and mounted flush with an 8.4 m long wooden walkway. The video and GRF data were used to identify key events during the gait cycle, such as heel strike. Fluoroscopic images of the ACLR and contralateral knees were acquired using a mobile biplane X‐ray (MoBiX) imaging system [15] sampling at 200 frames/s. The X‐ray units were operated in continuous mode (110 kV, 13.1 mA) with an inter‐beam angle of ~60° and an X‐ray source to image intensifier distance of ~1.4 m. Each participant was exposed to a maximum of 0.74 mSv from the CT scan and biplane X‐ray imaging.

### 2.4 Calculation of Knee Kinematics

**Figure 1:**
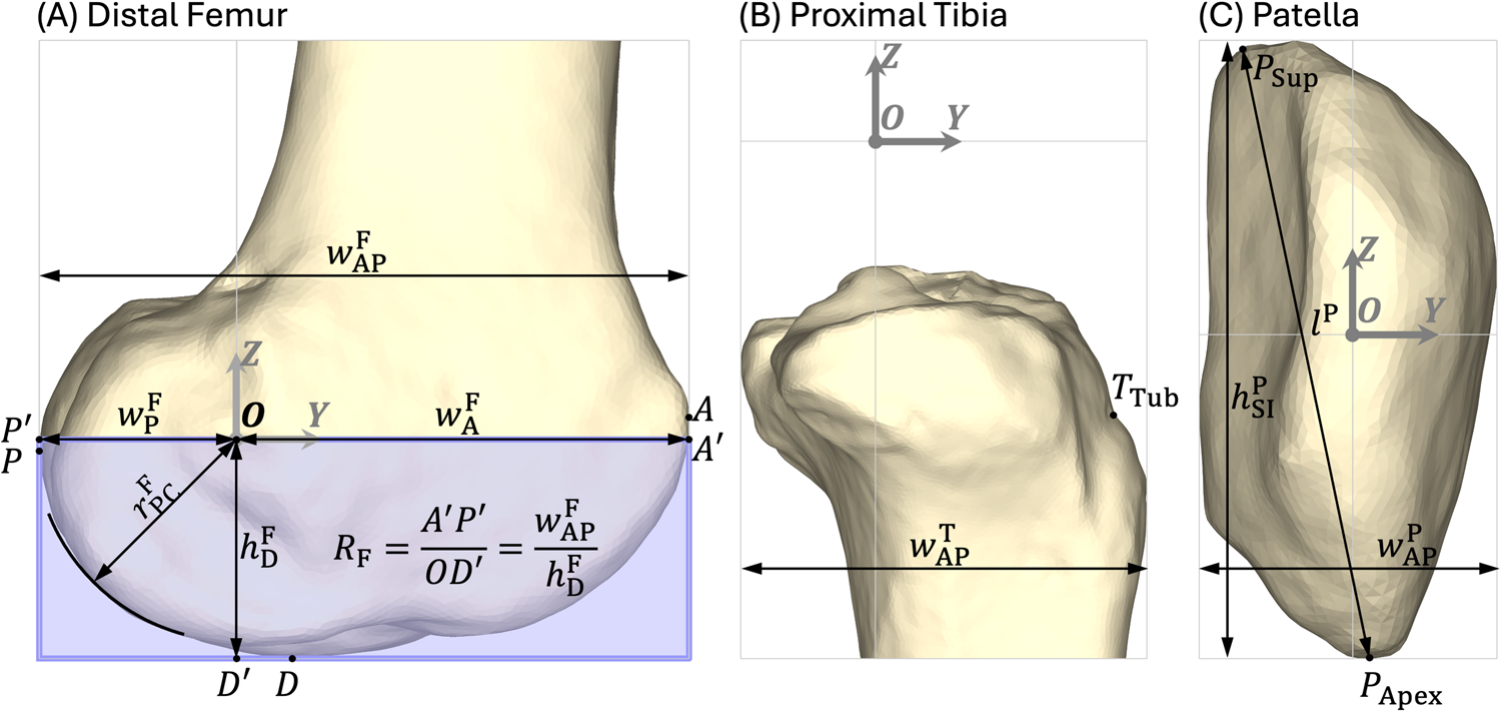
Coordinate systems and dimensions defined for the distal femur, proximal tibia, and patella of the right knee of a representative ACLR participant. The origin, *Y*‐axis, and *Z*‐axis of each bone are marked with O, Y, and Z, respectively, whereas the *X*‐axis (not shown) points out of the plane of the figure and laterally for each bone. Detailed definitions of the coordinate systems are given by Gray et al. [10]. (A) Distal femur: Points A, P, and D are the most anterior, most posterior, and most distal points, respectively, on the distal femur. A′ and P′ are the projections of points A and P onto the *Y*‐axis, while D′ is the projection of point D onto the *Z*‐axis. The anterior‐posterior width ($w_{\text{AP}}^{F}$) of the distal femur was measured as the distance AP projected onto the *Y*‐axis, that is, the distance $A^{\prime }P\prime$. The medial‐lateral width ($w_{\text{ML}}^{F}$, not shown) of the distal femur, that is, the femoral bicondylar width, was measured as the distance between the most medial and most lateral points projected onto the *X*‐axis. The distances OA′ ($w_{A}^{F}$) and OP′ ($w_{P}^{F}$) and the height OD′ ($h_{D}^{F}$) represent the anterior width, posterior width, and distal height of the femur, respectively, measured from the origin O. The radius of the posterior femoral condyles ($r_{\text{PC}}^{F}$) was also measured. The flatness of the blue box was used to visualize the flatness of the femoral condyle. A ratio $R_{F},$ defined as the length divided by the height of the blue box (i.e., $R_{F}=A\prime P\prime /\mathrm{OD}\prime =w_{\text{AP}}^{F}/h_{D}^{F})$, was introduced to quantify the flatness. (B) Proximal tibia: The insertion of the patellar tendon on the tibial tuberosity was identified as the most superior and posterior point, $T_{\text{Tub}}$, on the tibial tuberosity [20]. For both the proximal tibia and patella, the anterior‐posterior width ($w_{\text{AP}}^{T}$ and $w_{\text{AP}}^{P}$) was measured as the distance between the most anterior and most posterior points projected onto the *Y*‐axis, while the medial‐lateral width ($w_{\text{ML}}^{T}$ and $w_{\text{ML}}^{P}$, not shown) was measured as the distance between the most medial and most lateral points projected onto the *X*‐axis. (C) Patella: Patellar length ($l^{P}$) was defined as the distance between the apex of the patella, $P_{\text{Apex}}$, and the most posterior superior point, $P_{\text{Sup}}$, on the patellar bone model in the sagittal plane [20]. The apex of patella ($P_{\text{Apex}}$) was identified as the most inferior point on patella. The length of the patellar tendon was measured as the distance between points $P_{\text{Apex}}$ and $T_{\text{Tub}}$ in 3D space. Additionally, the height of the patella ($h_{\text{SI}}^{P}$) was measured as the distance between the most superior and most inferior points projected onto the *Z*‐axis.

6‐DOF patellofemoral and tibiofemoral joint kinematics for the ACLR knee, contralateral knee, and healthy knee were calculated from pose‐estimation by combining the volumetric bone models and biplane X‐ray images using custom MATLAB software (MathWorks, Natick, MA, USA). Biplane X‐ray data were processed at 50 Hz, filtered using a fourth‐order, low‐pass Butterworth filter with a cut‐off frequency of 10 Hz, and then resampled to 201 time points over one gait cycle. The anatomical coordinate system (Figure 1) used to define the relative positions and orientations of the femur, tibia, and patella is described by Gray et al. [10]. The accuracy of the MoBiX imaging system in measuring 3D dynamic motion of the intact knee during a simulated gait cycle was reported previously: maximum RMS errors were 0.78 mm and 0.77° for translations and rotations of the tibia relative to the femur [15], and 0.37 mm and 1.46° for translations and rotations of the patella relative to the femur [21].

### 2.5 Measurement of Knee Bone Size and Patellar Tendon Length

To facilitate the interpretation of the kinematic measurements, we also measured the size of each knee bone and the length of the patellar tendon. The dimensions of the distal femur, proximal tibia, and patella were determined from the volumetric models generated from the CT scans (Figure 1). The shape of the distal femur in the sagittal plane was characterized by the ratio $R_{F}$, defined as the anterior‐posterior width of the distal femur divided by the distance between the most distal point and the origin of the distal femur along the proximal‐distal direction (i.e., base length of the blue rectangle in Figure 1 divided by its height). A larger value of $R_{F}$ was indicative of a more elongated and flatter profile of the femoral condyles in the sagittal plane.

Patellar tendon length was measured from the biplane X‐ray data acquired for level walking and was found by estimating the distance between the attachment sites of the patellar tendon on the tibial tuberosity and the apex of the patella at each time point (Figure 1B,C). The length of the patellar tendon was taken as the mean of the measurements obtained for the stance phase (heel‐strike to toe‐off) because patellar tendon length was reasonably constant during this period, indicating that the patellar tendon was then taut (see Figure S1). We also calculated the Insall–Salvati ratio [22] for each knee by dividing the length of the patellar tendon by the length of the patella ($l^{P}$ in Figure 1C).

### 2.6 Statistical Analysis

The six translations of the patella and tibia relative to the femur were normalized using the ratio of the femoral bicondylar width measured for each knee to the mean femoral bicondylar width calculated for all 10 healthy knees (81.7 mm). Mean patellofemoral and tibiofemoral kinematics over one gait cycle were calculated for each knee and each activity. Means and standard deviations were calculated for each parameter (i.e., 6‐DOF patellofemoral and tibiofemoral kinematics, Insall–Salvati ratio, and the dimensions of the distal femur, proximal tibia, and patella) across all participants in each of the three groups. Paired *t*‐tests were performed to identify differences in all parameters between the ACLR knee and contralateral knee, whereas independent *t*‐tests were performed when comparing the ACLR knee and contralateral knee to the healthy knee. The significance level was set to *p* < 0.05.

## 3 Results

The mean difference in walking speed between the ACLR and healthy participants was within 0.1 m/s for both activities (see Table S1).

### 3.1 Patellofemoral Kinematics

**Figure 2:**
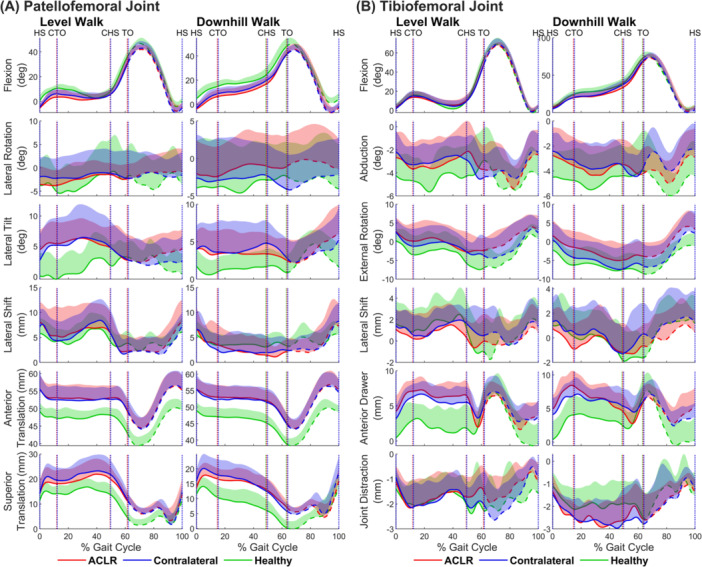
Six‐degree‐of‐freedom patellofemoral (A) and tibiofemoral (B) joint kinematics describing the rotations (rows 1–3) and translations (rows 4–6) of the patella and tibia with respect to the femur in level walking and downhill walking for the ACLR (red), contralateral (blue), and healthy (green) knees. The mean and standard deviation calculated across all participants in each group are plotted over the entire gait cycle. The solid and dashed lines represent the mean during the stance and swing phases of the gait cycle, respectively. The shaded areas represent one standard deviation from the mean. The vertical dotted lines indicate gait events: HS, heel‐strike; CTO, contralateral toe‐off; CHS, contralateral heel‐strike; and TO, toe‐off.

Patellofemoral joint motion was similar between the ACLR and contralateral knees in both activities, whereas significant differences were revealed when comparisons were made to the healthy knee, especially for the three sagittal‐plane movements of the patella: flexion, superior translation, and anterior translation (Figure 2A). In level walking, mean patellar flexion over the gait cycle was 4.5° less (*p* = 0.004), mean superior translation was 4.4 mm greater (*p* = 0.003), and mean anterior translation was 5.6 mm greater (*p* < 0.001) in the ACLR knee compared to the healthy knee (Table 1, panel A). Similar differences were observed in downhill walking, where mean patellar flexion was 7.0° less (*p* < 0.001), mean superior translation was 5.0 mm greater (*p* < 0.001), and mean anterior translation was 6.3 mm greater (*p* < 0.001) in the ACLR knee compared to the healthy knee (Table 1, panel B).

**Table 1:** Mean six‐degree‐of‐freedom patellofemoral and tibiofemoral joint kinematics calculated over one cycle of level walking (panels A and C) and downhill walking (panels B and D) for the ACLR knee, contralateral knee, and healthy knee.

	Mean ± Standard deviation			ACLR vs. Contralateral			ACLR vs. Healthy			Contralateral vs. Healthy		
	ACLR	Contra.	Healthy	Dif.	95% CI	*p*	Dif.	95% CI	*p*	Dif.	95% CI	*p*
**(A) Patellofemoral joint—Level walking**												
Lateral shift	4.8 ± 3.7	4.6 ± 3.0	4.4 ± 2.2	0.3 ± 2.1	−0.9 to 1.4	0.641	0.5	−2.2 to 3.2	0.724	0.2	−2.1 to 2.5	0.858
Anterior translation	51.9 ± 3.5	51.6 ± 3.5	46.2 ± 2.3	0.2 ± 2.0	−0.9 to 1.3	0.663	5.6	3.0 to 8.2	**< 0.001** *	5.4	2.8 to 8.0	**< 0.001** *
Superior translation	14.4 ± 3.5	15.2 ± 3.9	9.9 ± 2.7	−0.8 ± 2.9	−2.4 to 0.8	0.303	4.4	1.7 to 7.2	**0.003** *	5.3	2.3 to 8.2	**0.001** *
Flexion	11.3 ± 3.6	12.1 ± 3.8	15.8 ± 3.2	−0.8 ± 3.3	−2.6 to 1.1	0.378	−4.5	−7.4 to −1.6	**0.004** *	−3.7	−6.7 to −0.6	**0.020** *
Lateral rotation	−2.0 ± 2.8	−1.7 ± 4.3	−3.1 ± 5.6	−0.3 ± 4.1	−2.5 to 2.0	0.789	1.1	−2.4 to 4.6	0.525	1.4	−2.7 to 5.5	0.495
Lateral tilt	4.5 ± 2.3	4.0 ± 3.4	1.7 ± 2.2	0.5 ± 2.7	−1.0 to 2.0	0.507	2.8	0.9 to 4.7	**0.005** *	2.4	−0.2 to 4.9	0.067
**(B) Patellofemoral joint—Downhill walking**												
Lateral shift	3.0 ± 3.1	3.0 ± 3.0	3.9 ± 1.9	−0.0 ± 2.0	−1.2 to 1.1	0.934	−1.0	−3.3 to 1.3	0.394	−0.9	−3.2 to 1.3	0.410
Anterior translation	51.6 ± 3.2	51.3 ± 3.7	45.3 ± 2.4	0.3 ± 1.9	−0.7 to 1.4	0.494	6.3	3.8 to 8.8	**< 0.001** *	5.9	3.1 to 8.7	**< 0.001** *
Superior translation	11.7 ± 2.4	12.3 ± 3.3	6.7 ± 3.3	−0.6 ± 2.9	−2.3 to 1.1	0.479	5.0	2.7 to 7.4	**< 0.001** *	5.6	2.8 to 8.5	**< 0.001** *
Flexion	15.2 ± 4.2	16.2 ± 4.1	22.2 ± 4.0	−0.9 ± 3.5	−3.0 to 1.1	0.334	−7.0	−10.5 to −3.4	**< 0.001** *	−6.0	−9.5 to −2.6	**0.002** *
Lateral rotation	−1.2 ± 3.8	−2.9 ± 4.5	−3.3 ± 5.4	1.7 ± 4.2	−0.7 to 4.1	0.156	2.2	−1.7 to 6.1	0.260	0.5	−3.7 to 4.7	0.815
Lateral tilt	3.5 ± 2.2	3.7 ± 2.5	1.9 ± 1.2	−0.2 ± 2.5	−1.6 to 1.2	0.771	1.6	0.0 to 3.2	**0.048** *	1.8	0.0 3.6	**0.050** *
**(C) Tibiofemoral joint—Level walking**												
Lateral shift	0.4 ± 2.0	0.9 ± 1.5	0.8 ± 2.0	−0.5 ± 1.0	−1.1 to 0.1	0.078	−0.4	−2.1 to 1.3	0.646	0.1	−1.3 to 1.6	0.856
Anterior drawer	5.2 ± 1.6	5.1 ± 1.7	2.3 ± 2.5	0.1 ± 2.2	−1.1 to 1.4	0.799	3.0	1.2 to 4.7	**0.002** *	2.8	1.1 to 4.6	**0.003** *
Joint distraction	−1.6 ± 0.7	−1.8 ± 0.6	−1.9 ± 0.8	0.2 ± 0.9	−0.3 to 0.7	0.360	0.3	−0.3 to 0.9	0.354	0.1	−0.5 to 0.7	0.799
Flexion	22.1 ± 3.8	22.4 ± 4.0	22.4 ± 3.7	−0.3 ± 3.9	−2.5 to 1.9	0.775	−0.3	−3.5 to 2.8	0.825	0.0	−3.3 to 3.3	0.976
Abduction	−3.4 ± 2.1	−3.3 ± 1.4	−4.5 ± 2.2	−0.1 ± 1.8	−1.1 to 0.9	0.838	1.1	−0.7 to 2.9	0.222	1.2	−0.3 to 2.7	0.105
External rotation	0.0 ± 3.5	−1.2 ± 4.1	−3.3 ± 3.3	1.3 ± 3.8	−0.9 to 3.4	0.224	3.4	0.5 to 6.3	**0.024** *	2.1	−1.1 to 5.4	0.191
**(D) Tibiofemoral joint—Downhill walking**												
Lateral shift	−0.2 ± 1.7	0.4 ± 1.8	0.0 ± 1.9	−0.6 ± 1.0	−1.2 to −0.1	**0.032** *	−0.3	−1.8 to 1.3	0.738	0.4	−1.2 to 1.9	0.624
Anterior drawer	5.6 ± 1.9	5.4 ± 1.7	3.0 ± 2.5	0.1 ± 2.6	−1.3 to 1.6	0.833	2.6	0.7 to 4.5	**0.009** *	2.4	0.7 to 4.2	**0.008** *
Joint distraction	−2.2 ± 0.7	−2.2 ± 0.9	−1.7 ± 0.8	0.0 ± 0.8	−0.4 to 0.5	0.875	−0.5	−1.1 to 0.2	0.138	−0.5	−1.2 to 0.2	0.171
Flexion	31.0 ± 4.5	31.7 ± 5.0	34.4 ± 4.7	−0.7 ± 4.4	−3.3 to 1.8	0.532	−3.5	−7.4 to 0.4	0.079	−2.7	−6.9 to 1.4	0.189
Abduction	−3.8 ± 1.8	−3.4 ± 1.2	−4.8 ± 2.3	−0.4 ± 1.5	−1.3 to 0.5	0.313	1.0	−0.7 to 2.7	0.237	1.4	−0.0 to 2.9	0.054
External rotation	−1.9 ± 3.5	−4.2 ± 4.9	−5.7 ± 3.2	2.3 ± 3.1	0.5 to 4.1	**0.017** *	3.8	0.9 to 6.8	**0.012** *	1.6	−2.1 to 5.2	0.383

*Note:* Units: translations are in mm; rotations are in degrees. Abbreviations: CI, confidence interval; Contra., contralateral; Dif., difference.

* Bold type indicates *p* < 0.05 for *t*‐tests.

Patellar motion was also significantly different in the contralateral knee relative to the healthy knee in both activities (Figure 2A). In level walking, mean patellar flexion over the gait cycle was 3.7° less (*p* = 0.020), mean superior translation was 5.3 mm greater (*p* = 0.001), and mean anterior translation was 5.4 mm greater (*p* < 0.001) in the contralateral knee compared to the healthy knee (Table 1, panel A). In downhill walking, mean patellar flexion was 6.0° less (*p* = 0.002), mean superior translation was 5.6 mm greater (*p* < 0.001), and mean anterior translation was 5.9 mm greater (*p* < 0.001) in the contralateral knee compared to the healthy knee (Table 1, panel B). There were no significant differences in mean patellofemoral kinematics over the gait cycle between the ACLR knee and contralateral knee in both activities.

### 3.2 Tibiofemoral Kinematics

Significant differences were observed in external tibial rotation and anterior tibial translation between the three groups (Figure 2B, row 3 “external rotation” and row 5 “anterior drawer”). In level walking, mean external tibial rotation over the gait cycle was 1.3° greater in the ACLR knee compared to the contralateral knee (*p* = 0.224) (Table 1, panel C). In downhill walking, mean external tibial rotation over the gait cycle was 2.3° greater for the ACLR knee than the contralateral knee (*p* = 0.017) (Table 1, panel D). Mean external tibial rotation over the gait cycle was 3.4° greater in the ACLR knee compared to the healthy knee in level walking (*p* = 0.024) and 3.8° greater in downhill walking (*p* = 0.012). There were no significant differences in mean external tibial rotation between the contralateral knee and healthy knee in both activities.

Anterior tibial translation was significantly greater in the ACLR and contralateral knees compared to the healthy knee in both activities when the knee was flexed < 30° (Figure 2B, rows 1 and 5). In level walking, mean anterior tibial translation over the gait cycle was 3.0 mm greater in the ACLR knee (*p* = 0.002) and 2.8 mm greater in the contralateral knee (*p* = 0.003) compared to the healthy knee (Table 1, panel C). In downhill walking, mean anterior tibial translation was 2.6 mm greater in the ACLR knee (*p* = 0.009) and 2.4 mm greater in the contralateral knee (*p* = 0.008) compared to the healthy knee (Table 1, panel D). There were no significant differences in anterior tibial translation between the ACLR knee and contralateral knee in both activities.

### 3.3 Knee Bone Size and Patellar Tendon Length

There were no significant differences in knee bone size between the ACLR and contralateral knees, whereas two significant differences were identified in the size of the distal femur when comparing the ACLR and contralateral knees to the healthy knee (Table 2, panels A–C). First, the distance OA′ (Figure 1A), defined as the anterior‐posterior distance from the center of the posterior femoral condyle (point O) to the most anterior landmark on the distal femur (point A′), was 3.7 mm greater in the ACLR knee (*p* = 0.013) and contralateral knee (*p* = 0.012) compared to the healthy knee (Table 2, panel A). Second, the ratio $R_{F}$ (Figure 1A), which quantified the elongation and flatness of the femoral condyles, was greater in the ACLR knee and contralateral knee compared to the healthy knee (*p* = 0.002 and *p* = 0.014, respectively; Table 2, panel A).

**Table 2:** Measurements of the distal femur, proximal tibia, patella, and patellar tendon for the ACLR knee, contralateral knee, and healthy knee.

	Mean ± Standard deviation			ACLR vs. Contralateral			ACLR vs. Healthy			Contralateral vs. Healthy		
	ACLR	Contra.	Healthy	Dif.	95% CI	*p*	Dif.	95% CI	*p*	Dif.	95% CI	*p*
**(A) Femur**												
ML width ($w_{\text{ML}}^{F}$)	81.1 ± 5.6	80.6 ± 5.7	81.7 ± 7.0	0.5 ± 0.9	0.0 to 1.0	0.038*	−0.7	−5.9 to 4.5	0.795	−1.2	−6.4 to 4.1	0.650
AP width ($w_{\text{AP}}^{F}$)	67.0 ± 4.8	66.8 ± 4.3	64.5 ± 4.0	0.2 ± 1.0	−0.3 to 0.8	0.376	2.6	−1.2 to 6.4	0.173	2.3	−1.2 to 5.9	0.182
OA′ ($w_{A}^{F}$)	44.7 ± 3.7	44.7 ± 3.6	41.0 ± 2.7	0.0 ± 1.6	−0.8 to 0.9	0.908	3.7	0.9 to 6.6	**0.013** *	3.7	0.9 to 6.4	**0.012** *
OP′ ($w_{P}^{F}$)	22.3 ± 1.8	22.1 ± 2.5	23.4 ± 1.7	0.2 ± 1.6	−0.7 to 1.1	0.653	−1.1	−2.6 to 0.4	0.139	−1.3	−3.2 to 0.6	0.160
OD′ ($h_{D}^{F}$)	24.1 ± 1.9	23.9 ± 2.3	24.7 ± 1.7	0.2 ± 1.3	−0.5 to 0.9	0.569	−0.6	−2.1 to 0.9	0.427	−0.8	−2.6 to 1.0	0.366
Condyle radius ($r_{\mathrm{PC}}^{F}$)	22.6 ± 1.9	22.4 ± 2.7	23.9 ± 1.8	0.2 ± 1.8	−0.8 to 1.2	0.697	−1.3	−2.9 to 0.2	0.094	−1.5	−3.5 to 0.5	0.136
Ratio $R_{F}$ ($w_{\text{AP}}^{F}/h_{D}^{F}$)	2.78 ± 0.13	2.80 ± 0.21	2.61 ± 0.11	−0.02 ± 0.14	−0.10 to 0.06	0.551	0.17	0.07 to 0.28	**0.002** *	0.20	0.04 to 0.35	**0.014** *
**(B) Tibia**												
ML width ($w_{\text{ML}}^{T}$)	76.5 ± 5.6	76.2 ± 5.7	76.2 ± 6.1	0.3 ± 0.8	−0.2 to 0.7	0.235	0.3	−4.6 to 5.2	0.909	0.0	−4.9 to 5.0	0.997
AP width ($w_{\text{AP}}^{T}$)	62.2 ± 4.6	62.3 ± 4.8	61.7 ± 5.5	−0.1 ± 1.6	−1.0 to 0.8	0.848	0.6	−3.7 to 4.8	0.788	0.6	−3.7 to 4.9	0.764
**(C) Patella**												
ML width ($w_{\text{ML}}^{P}$)	43.0 ± 3.7	43.0 ± 3.5	43.0 ± 4.8	0.0 ± 1.0	−0.6 to 0.6	0.987	0.0	−3.6 to 3.5	0.977	−0.1	−3.5 to 3.4	0.974
AP width ($w_{\text{AP}}^{P}$)	21.6 ± 1.2	21.4 ± 1.1	21.5 ± 1.8	0.2 ± 0.6	−0.1 to 0.6	0.212	0.1	−1.2 to 1.3	0.879	−0.1	−1.3 to 1.1	0.833
SI height ($h_{\text{SI}}^{P}$)	42.0 ± 3.3	41.6 ± 2.8	40.9 ± 3.7	0.4 ± 1.1	−0.2 to 1.1	0.160	1.1	−1.8 to 4.1	0.426	0.7	−1.9 to 3.4	0.584
Length $\left(l^{P}\right)$	42.6 ± 3.2	42.1 ± 2.7	41.4 ± 3.8	0.5 ± 1.1	−0.2 to 1.1	0.141	1.2	−1.7 to 4.1	0.410	0.7	−1.9 to 3.4	0.577
**(D) Patellar tendon**												
Length ($P_{\text{Apex}}T_{\text{Tub}}$)	51.9 ± 4.9	50.9 ± 5.8	42.5 ± 3.1	1.0 ± 2.2	−0.2 to 2.3	0.093	9.4	5.8 to 13.0	**< 0.001** *	8.4	4.2 to 12.6	**< 0.001** *
Insall–Salvati ratio	1.22 ± 0.12	1.21 ± 0.14	1.03 ± 0.06	0.01 ± 0.08	−0.03 to 0.06	0.518	0.19	0.11 to 0.28	**< 0.001** *	0.18	0.09 to 0.28	**< 0.001** *

*Note:* See Figure 1 for details of each measurement. Units: All measurements other than the two ratios (i.e., ratio $R_{F}$ and Insall–Salvati ratio) are in mm. Abbreviations: CI, confidence interval; Contra., contralateral; Dif., difference.

* Bold type indicates *p* < 0.05 for *t*‐tests.

The patellar tendon was on average 8.9 mm longer (*p* < 0.001) in both the ACLR and contralateral knees compared to the healthy knee (Table 2, panel D). Patellar tendon lengths for the ACLR and contralateral knees were, respectively, 51.9 ± 4.9 and 50.9 ± 5.8 mm compared to 42.5 ± 3.1 mm for the healthy knee. Insall–Salvati ratios for the ACLR, contralateral, and healthy knees were, respectively, 1.22 ± 0.12, 1.21 ± 0.14, and 1.03 ± 0.06 (*p* < 0.001 for both ACLR vs. healthy and contralateral vs. healthy). There were no significant differences in patellar tendon length between the ACLR and contralateral knees (Table 2, panel D).

## 4 Discussion

We used mobile biplane X‐ray imaging to measure 6‐DOF patellofemoral and tibiofemoral joint motion in both knees of ACLR participants during level walking and downhill walking, and compared the results to those for healthy knees (controls) [16]. Significant differences were observed in all three sagittal‐plane movements of the patella relative to the femur for both the ACLR and contralateral knees compared to the healthy knee in both activities (Table 1, panels A and B). Thus, our first hypothesis regarding no differences in patellofemoral kinematics across the three groups was rejected. At the tibiofemoral joint, mean external tibial rotation over the gait cycle was significantly greater (by ~3°) for the ACLR knee compared to the healthy knee in both activities and for the ACLR knee compared to the contralateral knee in downhill walking but not level walking (Table 1, panels C and D), hence our second hypothesis was partially supported.

**Figure 3:**
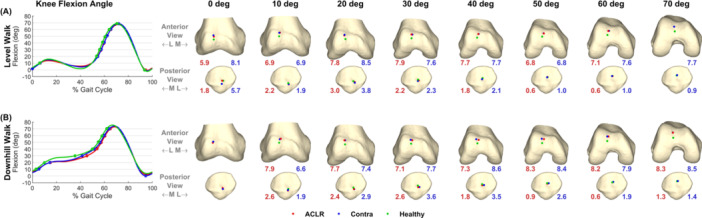
Cartilage contact centers on the femoral trochlea (top row) and on the patellar facet (bottom row) for the ACLR (red), contralateral (blue), and healthy (green) knees measured during level walking (A) and downhill walking (B). Data are shown for increments of 10° of the knee (tibiofemoral) flexion angle over one gait cycle. The red, blue, and green dots on the femoral trochlea and patella represent the mean contact center calculated across all participants in each group. The red number at the bottom of each distal femur model and each patella model specifies the distance between the mean contact centers measured on the ACLR knee and healthy knee, whereas the blue number specifies the distance between the mean contact centers measured on the contralateral knee and healthy knee. For example, at 10° of knee flexion, the distance between the contact centers on the femoral trochlea of the ACLR knee and healthy knee was 6.9 mm for level walking and 7.9 mm for downhill walking, whereas the distance between the contact centers on the femoral trochlea of the contralateral knee and healthy knee was 6.9 mm for level walking and 6.6 mm for downhill walking. The panels on the left‐hand side of the figure specify the time instants of the gait cycle that correspond to the locations of the patellofemoral joint contact centers shown on the right‐hand side. L, lateral; M, medial.

Mean superior translation of the patella was greater in the ACLR knee and contralateral knee compared to the healthy knee in both activities (Figure 2 and Table 1), indicating an elevated position of the patella in both knees of the ACLR participants. To further elucidate this result, we calculated the locations of cartilage contact at the patellofemoral joint in all three groups for each activity (see Supporting Information, Section S2, for associated methods). In both activities, at the same tibiofemoral (knee) flexion angle, the cartilage contact centers were higher on the femoral trochlea in the ACLR and contralateral knees compared to the healthy knee (Figure 3). The 3D distances between the mean contact centers on the femoral trochlea in the ACLR knee vs. healthy knee ranged from 5.9 to 8.3 mm (mean = 7.5 mm), while those for the contralateral knee vs. healthy knee ranged from 6.6 to 8.6 mm (mean = 7.7 mm). These findings directly confirm that the patella articulated higher on the femoral trochlea in the ACLR and contralateral knees compared to the healthy knee.

**Figure 4:**
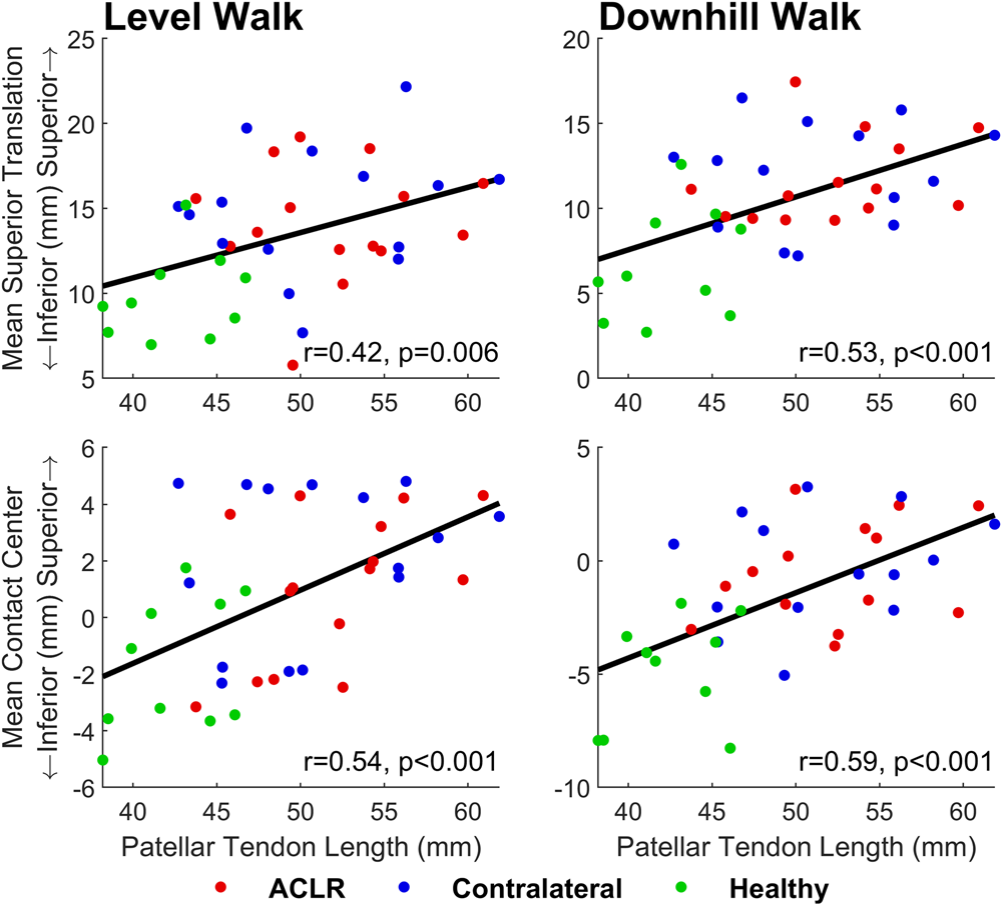
Mean patellar superior translation (top row) and mean location of the patellofemoral joint contact center on the femoral trochlea (bottom row) calculated over one gait cycle for level walking (left column) and downhill walking (right column) and plotted against mean patellar tendon length. The colored dots signify measurements obtained from each ACLR knee (red), contralateral knee (blue), and healthy knee (green). The black line represents the least squares regression line fitted to all data points, with the Pearson correlation coefficient (*r*) and *p* value displayed at the bottom‐right of each panel.

A higher riding patella was explained by a significantly longer patellar tendon in both the ACLR knee and contralateral knee compared to the healthy knee (Figure 4). Mean patellar tendon length was 9.4 mm (22%) longer in the ACLR knee and 8.4 mm (20%) longer in the contralateral knee compared to the healthy knee (Table 2, panel D). In addition, the patellar tendon was taut from 0% to 80% of the gait cycle in all three groups (Figure S1), and during this period, superior translation of the patella was significantly greater in the ACLR and contralateral knees compared to the healthy knee (Figure 2, row 6, column 1), further supporting that a longer patellar tendon led to a higher riding patella. The mean Insall–Salvati ratio was 1.22 for the ACLR knee (range: 1.03–1.50) and 1.21 for the contralateral knee (range: 0.99–1.40). An Insall–Salvati ratio ≥ 1.20 indicates the presence of patella alta, a clinical condition associated with patellar instability and anterior knee pain [20, 23]. Nine of the 15 ACLR participants had patella alta in at least one of their knees, and five of these participants had patella alta in both knees. Overall, 14 of the 30 knees (47%) tested from the ACLR group had patella alta. The mean Insall–Salvati ratio for the healthy control group was 1.03 (range: 0.93–1.15). We also calculated the modified Insall–Salvati [24] and Blackburne‐Peel ratios [25] and found that these indices identified fewer knees with patella alta in the ACLR group (6/30 [20%] for the modified Insall–Salvati ratio; and 7/30 [23%] for the Blackburne–Peel ratio) (Table S3). While these results may cast some uncertainty regarding the incidence of patella alta among the ACLR participants, our kinematic data clearly show that the vertical position of the patella was considerably higher in the ACLR and contralateral knees compared to the healthy knee in both activities. Previous studies have pointed to a high‐riding patella as a possible risk factor for ACL injury [26, 27]. Degnan et al. [26] found that patellar tendon length and the Insall–Salvati ratio were significantly higher in children who had experienced an ACL tear, while Güven et al. [27] found a significantly higher Insall–Salvati ratio in adult males with an ACL tear. Future studies should investigate the association between patellar height and the incidence of ACL injury.

Another prominent feature of ACLR gait was increased external tibial rotation in the ACLR knee relative to the contralateral knee and healthy knee in both activities (Figure 2B). Mean external tibial rotation was 1.3°–3.8° greater in the ACLR knee than in the contralateral knee and healthy knee during gait (Table 1, panels C and D). Increased external tibial rotation in the ACLR knee relative to the contralateral knee has been reported for the stance phase of walking [6, 7], which may result from over‐tensioning of the ACL graft. Brady et al. [28] performed ACL reconstructions in 12 cadaveric knees, finding that an increase in initial graft tension reduced the amount of internal tibial rotation (increased external rotation) relative to the ACL‐intact knee at all flexion angles. At full extension, an initial graft tension of 90 N caused the tibia to rotate externally by ~2° relative to the intact knee [28]. In the present study, the graft was attached slightly distal to the anatomical site on the femur and was tensioned with the knee at full extension. Initial graft tension was not measured, but if it was say 90 N (~20 lbs), then as the femoral condyles elongate and flatten over time, as observed in our ACLR participants (see Figure 1 and Table 2), graft tension would increase further. The orientation of the ACL graft in the transverse plane is defined by its origin posteriorly on the medial side of the lateral femoral condyle and its insertion anteriorly and slightly medial to the central axis of the tibial plateau. A tight ACL graft would therefore induce a laterally directed force on the tibia, causing it to rotate externally.

Anterior tibial translation was significantly greater (by ~5 mm) in the ACLR knee and contralateral knee compared to the healthy knee when the knee was flexed < 30° in both activities (Figure 2B, row 5), and may be explained by the difference in the shapes of the femoral condyles between the knees of the ACLR participants and healthy controls (Table 2, panel A). The ratio $R_{F}$ was significantly greater in the ACLR knee (*p* = 0.002) and contralateral knee (*p* = 0.014) compared to the healthy knee, indicating that the femoral condyles of the ACLR participants were more elongated and flatter when viewed in the sagittal plane. A flatter profile of the femoral condyle manifested as a greater distance OA′ (by 3.7 mm) in the ACLR knee (*p* = 0.013) and contralateral knee (*p* = 0.012) relative to the healthy knee. An increase in the distance OA′ shifted the origin of the femur posteriorly relative to the tibia and caused an offset of ~4 mm in anterior tibial translation. However, this result should be viewed with caution because measurements of tibiofemoral joint translations are known to depend on the choice of the joint coordinate system [29]. In Table S2 and Figures S2–S4 of the Supporting Information, we show how measurements of tibiofemoral translations can be affected by the location chosen for the origin of the femoral coordinate system. This choice in the location of the femoral origin would also affect the measured patellofemoral joint translations, but our main finding of a higher riding patella in the ACLR knee and contralateral knee compared to the healthy knee would not be altered, because this result was determined primarily by the difference in patellar tendon length between the ACLR participants and healthy controls.

Altered knee joint motion after ACLR may be associated with the development of knee OA [3, 4, 6]. Andriacchi et al. [3] proposed that ACL injury can shift the load‐bearing areas of cartilage at the knee to regions of thinner cartilage where infrequent but excessive levels of load occur. Our results show that a longer patellar tendon was responsible for a higher vertical position of the patella and an elevated location of joint contact on the femoral trochlea in both knees of the ACLR participants compared to healthy controls. The patellar tendon was on average 8.9 mm longer in the ACLR and contralateral knees compared to the healthy knee (Table 2, panel D), and the cartilage contact center was correspondingly 7.6 mm higher in the ACLR and contralateral knees relative to the healthy knee in both activities (Figures 3 and 4). An elevated location of joint contact on the trochlea may lead to joint overloading as higher and more frequent levels of joint load are imposed on regions of cartilage not adequately conditioned to withstand such loads. Additionally, an elevated location of joint contact may lead to joint underloading because regions of the trochlear cartilage that once articulated with the patella may now experience normal levels of joint load less frequently. Thus, a high‐riding patella may result in the femoral trochlear cartilage being overloaded as well as underloaded over the course of each gait cycle.

Aside from shifting the load‐bearing regions of cartilage, an elevated position of the patella may reduce patellofemoral joint contact area and drive the development of patellofemoral OA by increasing joint stress. Elevated joint stress is believed to contribute to cartilage degeneration (overloading) [30]. Ward et al. [23] measured patellofemoral contact area in subjects with patella alta and healthy controls as the knee was flexed from 0° (full extension) to 60° with the quadriceps contracted. At all flexion angles, subjects with patella alta had significantly less patellofemoral joint contact area compared to those with normal patellar positions (controls). Further, the patella alta group with reduced patellofemoral joint contact area demonstrated significantly higher patellofemoral joint stress during gait [31]. Our on‐going work involves using mobile biplane fluoroscopy and magnetic resonance imaging to measure patellofemoral joint contact area [32] after ACLR.

Our results suggest that the contralateral knee is likely not an appropriate control when assessing knee joint motion during gait after ACLR. The only consistent difference observed bilaterally in the knees of our ACLR participants was increased external tibial rotation in the ACLR knee relative to the contralateral knee, whereas significant differences in anterior tibial translation, external tibial rotation, patellar flexion, superior patellar translation, and anterior patellar translation were found between both these knees and the healthy knee. Others have also raised concerns regarding the use of the contralateral knee as a control [2]. We recommend the use of a separate control group comprised of well‐matched healthy individuals with no knee pain or history of lower‐limb pathology in cross‐sectional studies aimed at elucidating the effects of ACLR on knee cartilage health.

One potential limitation of our study is that the ACLR and control groups were not matched for activity level: the ACLR participants were physically active individuals who participated in competitive sports at least once a week, whereas the healthy participants were drawn from a pool of college students, most of whom did not regularly engage in sports. It is possible that the longer patellar tendons observed in the ACLR participants were induced by higher levels of physical activity. However, previous studies found patellar tendon lengths to be similar in athletes and non‐athletes; for example, Wiesinger et al. [33] reported no significant differences in patellar tendon lengths between elite ski‐jumpers, endurance runners, water polo players, and sedentary controls. A second potential limitation is that the ACLR participants were on average 6.2 cm taller than the controls (*p* = 0.182) which, instead of knee type (ACLR vs. contralateral vs. healthy), may have been responsible for the longer patellar tendons observed in the ACLR participants. To investigate this issue further, we performed a post hoc ANOVA with body height and weight introduced as co‐variates and found that knee type was still a factor (*p* < 0.001) significantly affecting patellar tendon length. In addition, previous studies found no correlation [34] or at most a weak correlation [35] between body height and patellar tendon length. Indeed, patellar tendon length has been shown to vary considerably even among individuals of the same height [36]. Finally, we are unable to establish whether a high‐riding patella observed in the ACLR participants existed before the ACL injury or resulted from the ACL injury and/or ACLR surgery. Measurements of patellar tendon length at different time points are needed to directly address this issue; for example, immediately after ACL injury, immediately after ACLR, and at some time after ACLR. We measured patellar tendon length for the ACLR participants at a single time point, between 6 and 15 months after ACLR. Longitudinal studies are required to determine the cause of a longer patellar tendon and a high‐riding patella in individuals who have undergone ACLR surgery.

In conclusion, the main finding of this study was that the patella articulated higher (by 7.6 mm) on the femoral trochlea in both the ACLR and contralateral knees compared to the healthy knee, due primarily to a longer patellar tendon (by 8.9 mm). A higher riding patella may contribute to the development of patellofemoral OA by shifting the load‐bearing areas on the femoral trochlea to regions of cartilage unaccustomed to load and leaving previously loaded regions underloaded.

## Author Contributions

M.G.P., H.A.G., A.G.C., K.M.C., and S.G. designed the study. M.G.P. obtained funding for the research. A.G.C. and S.G. performed subject recruitment. H.A.G. and S.G. collected the data. H.A.G., P.N.G., and S.G. processed the data. M.G.P., H.A.G., and S.G. analyzed and interpreted the data. M.G.P. and S.G. drafted the manuscript. All authors edited, revised, and approved the final submitted manuscript.

## Supporting information

Supporting information.
