# Supplementary material for: A High‐Riding Patella Is a Feature of Knee Joint Motion During Gait After ACL Reconstruction
Source: J Orthop Res. 2025 Mar 19;43(5):1024–34. doi: 10.1002/jor.26062 (PMC11982598; doi:10.1002/jor.26062)
Supplement: Supplementary file 1 — Supporting information. [file JOR-43-1024-s001.pdf]

SUPPLEMENTAL MATERIAL

A HIGH-RIDING PATELLA IS A FEATURE OF KNEE JOINT MOTION DURING GAIT AFTER ACL RECONSTRUCTION

Marcus G. Pandy, Hans A. Gray, Padma N. Ganapam, Adam G. Culvenor, Kay M. Crossley, Shanyuanye Guan

S1. Length of patellar tendon

For each participant, the length of the patellar tendon was first calculated for each time point during a level walking trial and was then taken as the mean of the length calculated during the stance phase. At each time point during level walking, the length of the patellar tendon was measured as the distance between the attachment point of the patellar tendon on the tibial tuberosity (T_{Tub} in Fig.1, panel B) and the apex of the patella (P_{Apex} in Fig.1, panel C). The length of the patellar tendon remained reasonably constant during the stance phase (Fig. S1), which indicated that the patellar tendon was taut during this period. Therefore, the length of the patellar tendon was taken as the mean length over the stance phase.

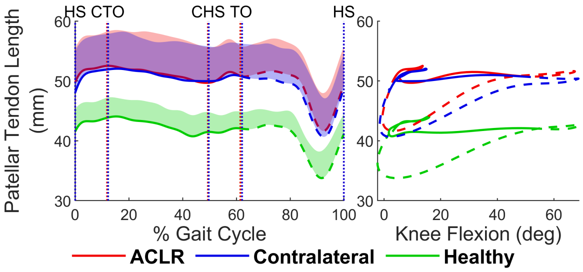

Supplementary Figure S1. Patellar tendon length measured in the ACLR (red), contralateral (blue), and healthy (green) knees plotted against the percentage of one gait cycle for level walking (left panel) and also plotted against the knee (tibiofemoral) flexion angle (right panel). The solid and dashed lines represent the mean during the stance and swing phases of the gait cycle, respectively. The shaded areas represent one standard deviation from the mean. The vertical dotted lines indicate gait events: HS, heel-strike; CTO, contralateral toe-off; CHS, contralateral heel-strike; and TO, toe-off.

S2. Calculation of the location of patellofemoral joint contact

The location of articular cartilage contact at the patellofemoral joint was calculated at each time point during the gait cycle for level walking and downhill walking. For each ACLR participant, MR scans (Achieva 3.0T, Philips; slice thickness = 0.7 mm; voxel size = 0.35 mm × 0.35 mm × 0.35 mm; proton-density-weighted turbo spin-echo; repetition time = 1300 msec; echo time = 27 msec; flip angle = 90°; echo train length = 64) of the ACLR knee and contralateral knee were acquired with the participant lying supine and the knee extended. Volumetric models of the cartilage on the distal femur and patella were created from the MR scans using 3D Slicer (Pieper *et al*., 2006), and were then aligned with the bone models using Geomagic Studio (3D Systems, Rockhill, SC). Articular contact was defined by the intersection of the cartilage layers lining the patella and the femoral trochlea. At each time point during the gait cycle, the relative pose (i.e., position and orientation) between the patellar and femoral cartilage models were determined by the relative pose between the bone models of the patella and femur measured using mobile biplane X-ray imaging. The patellar and femoral cartilage models were then represented by volumetric point clouds, and the points appearing in both cartilage point clouds determined the patellofemoral contact volume. The centroid of the cartilage contact volume was identified as the center of cartilage contact, which was then mapped to the closest surface triangle on the bone models of the patella and femur. Details of this procedure are given by Thomeer *et al*. (2022).

S3. Temporospatial gait parameters

Table S1. Temporospatial gait parameters measured during level walking (panel A) and downhill walking (panel B) for the ACLR knee, contralateral knee, and healthy knee.

	Mean ± Standard Deviation			ACLR vs Contralateral			ACLR vs Healthy			Contralateral vs Healthy		
	ACLR	Contra.	Healthy	Dif.	95% CI	P-val.	Dif.	95% CI	P-val.	Dif.	95% CI	P-val.
(A) Level Walking												
Walk Speed (m/s)	1.22±0.08	1.24±0.11	1.32±0.14	-0.02±0.10	-0.07 to 0.04	0.535	-0.09	-0.19 to -0.00	**0.045***	-0.08	-0.18 to 0.03	0.137
Stride Time (s)	1.20±0.10	1.20±0.10	1.10±0.10	0.00±0.07	-0.03 to 0.04	0.814	0.11	0.02 to 0.19	**0.014***	0.10	0.02 to 0.18	**0.021***
Cadence (steps/min)	100.6±8.7	100.9±8.2	110.3±9.3	-0.4±5.6	-3.5 to 2.8	0.812	-9.7	-17.2 to -2.2	**0.014***	-9.3	-16.6 to -2.1	**0.014***
Stride Length (m)	1.46±0.11	1.47±0.10	1.43±0.09	-0.01±0.06	-0.04 to 0.02	0.559	0.03	-0.05 to 0.12	0.460	0.04	-0.04 to 0.12	0.293
(B) Downhill Walking												
Walk Speed (m/s)	0.81±0.10	0.80±0.10	0.85±0.09	0.01±0.04	-0.02 to 0.04	0.406	-0.03	-0.11 to 0.05	0.436	-0.04	-0.12 to 0.04	0.298
Stride Time (s)	1.26±0.15	1.25±0.14	1.16±0.11	0.02±0.05	-0.01 to 0.05	0.149	0.11	-0.01 to 0.23	0.064	0.09	-0.02 to 0.20	0.109
Cadence (steps/min)	96.2±12.5	97.6±12.0	104.7±9.8	-1.4±3.8	-3.6 to 0.8	0.194	-8.4	-18.3 to 1.4	0.090	-7.0	-16.6 to 2.5	0.142
Stride Length (m)	1.02±0.04	0.99±0.04	0.97±0.02	0.03±0.04	0.00 to 0.05	**0.031***	0.05	0.02 to 0.08	**0.004***	0.02	-0.01 to 0.05	0.125

*Bold type indicates P < 0.05 for t tests.

Abbreviations: Contra., contralateral; Dif., difference; CI, confidence interval; P-val., p-value.

S4. Effect of joint coordinate system on six-degree-of-freedom tibiofemoral and patellofemoral joint kinematics

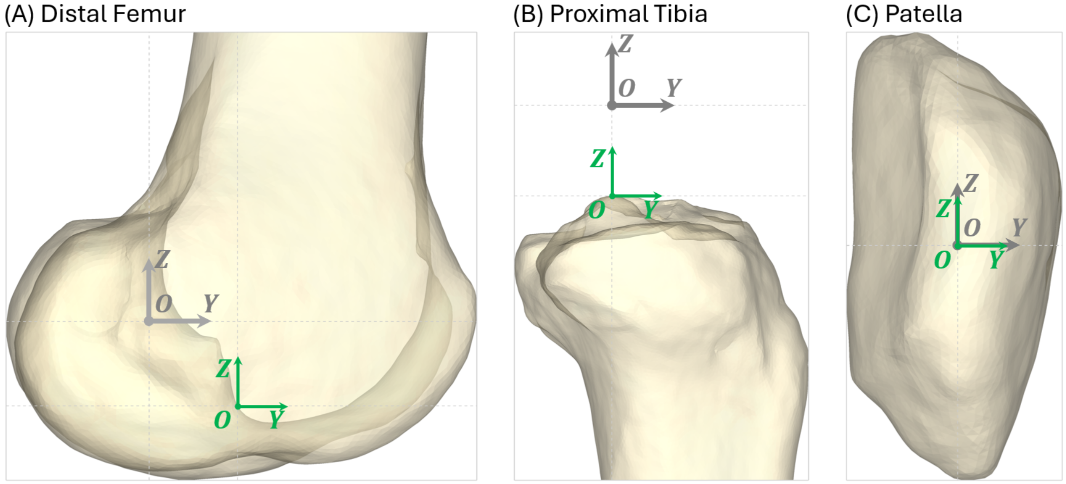

Supplementary Figure S2. Coordinate systems defined for the distal femur (panel A), proximal tibia (panel B), and patella (panel C) of the right knee of a representative ACLR participant. The origin, Y-axis, and Z-axis of each bone are marked with O, Y, and Z, respectively, whereas the X-axis (not shown) points out of the plane of the figure (laterally) for each bone. The gray-colored arrows and letters show the coordinate system defined by Gray *et al*. (2019), whereas green arrows and letters marked on the femur (panel A) and tibia (panel B) indicate the coordinate system defined by Grood and Suntay (1983). The patellar coordinate system (panel C) used to calculate patellofemoral joint kinematics for the Grood and Suntay coordinate system was identical with that defined by Gray *et al*. (2019).

Supplementary Table S2. Mean translations (in mm) at the patellofemoral and tibiofemoral joints calculated over one cycle of level walking (panels A and C) and downhill walking (panels B and D) for the ACLR knee, contralateral knee, and healthy knee. Kinematic parameters were defined using the joint coordinate system defined by Grood and Suntay (1983). These results should be compared with those given in Table 1 of the text to see how the translations calculated at the patellofemoral and tibiofemoral joints depend on the choice of the joint coordinate system.

	Mean ± Standard Deviation			ACLR vs Contralateral			ACLR vs Healthy			Contralateral vs Healthy		
	ACLR	Contra.	Healthy	Dif.	95% CI	P-val.	Dif.	95% CI	P-val.	Dif.	95% CI	P-val.
(A) Patellofemoral Joint – Level Walking												
Lateral Shift	4.8±3.7	4.6±3.0	4.4±2.2	0.3±2.1	-0.9 to 1.4	0.641	0.5	-2.2 to 3.2	0.724	0.2	-2.1 to 2.5	0.858
Anterior Translation	38.5±3.7	37.0±3.9	34.4±2.3	1.6±2.1	0.4 to 2.7	**0.012***	4.2	1.4 to 6.9	**0.004***	2.6	-0.3 to 5.5	0.074
Superior Translation	24.6±3.7	25.0±2.8	20.0±2.4	-0.4±2.7	-1.9 to 1.1	0.590	4.6	1.8 to 7.3	**0.002***	5.0	2.7 to 7.2	**<0.001***
(B) Patellofemoral Joint – Downhill Walking												
Lateral Shift	3.0±3.1	3.0±3.0	3.9±1.9	-0.0±2.0	-1.2 to 1.1	0.934	-1.0	-3.3 to 1.3	0.394	-0.9	-3.2 to 1.3	0.410
Anterior Translation	37.4±3.6	35.8±3.9	32.3±2.2	1.6±1.7	0.6 to 2.6	**0.004***	5.1	2.4 to 7.8	**<0.001***	3.5	0.7 to 6.3	**0.016***
Superior Translation	20.9±2.9	21.1±2.5	15.4±2.7	-0.2±2.2	-1.4 to 1.1	0.750	5.5	3.0 to 7.9	**<0.001***	5.7	3.4 to 7.9	**<0.001***
(C) Tibiofemoral Joint – Level Walking												
Lateral Shift	-0.7±1.8	-0.2±1.5	-0.7±1.9	-0.5±1.0	-1.1 to 0.0	0.063	0.0	-1.5 to 1.6	0.955	0.6	-0.8 to 1.9	0.413
Anterior Drawer	-9.2±2.3	-10.5±2.4	-10.1±3.6	1.2±2.5	-0.2 to 2.6	0.082	0.8	-1.6 to 3.3	0.500	-0.4	-2.9 to 2.1	0.745
Joint Distraction	10.3±2.3	10.8±1.8	9.7±1.1	-0.5±1.5	-1.3 to 0.4	0.238	0.6	-1.0 to 2.2	0.441	1.1	-0.2 to 2.4	0.102
(D) Tibiofemoral Joint – Downhill Walking												
Lateral Shift	-1.5±1.6	-0.8±1.7	-1.6±1.7	-0.7±0.9	-1.2 to -0.2	**0.007***	0.1	-1.3 to 1.6	0.867	0.9	-0.6 to 2.3	0.238
Anterior Drawer	-10.1±2.5	-11.1±2.5	-11.1±3.7	1.0±2.7	-0.5 to 2.6	0.178	1.0	-1.6 to 3.6	0.443	-0.1	-2.7 to 2.5	0.966
Joint Distraction	12.5±2.1	13.3±1.8	12.6±1.1	-0.8±1.5	-1.7 to 0.0	0.052	-0.1	-1.6 to 1.4	0.900	0.7	-0.6 to 2.1	0.262

*Bold type indicates P < 0.05 for t tests.

Abbreviations: Contra., contralateral; Dif., difference; CI, confidence interval; P-val., p-value.

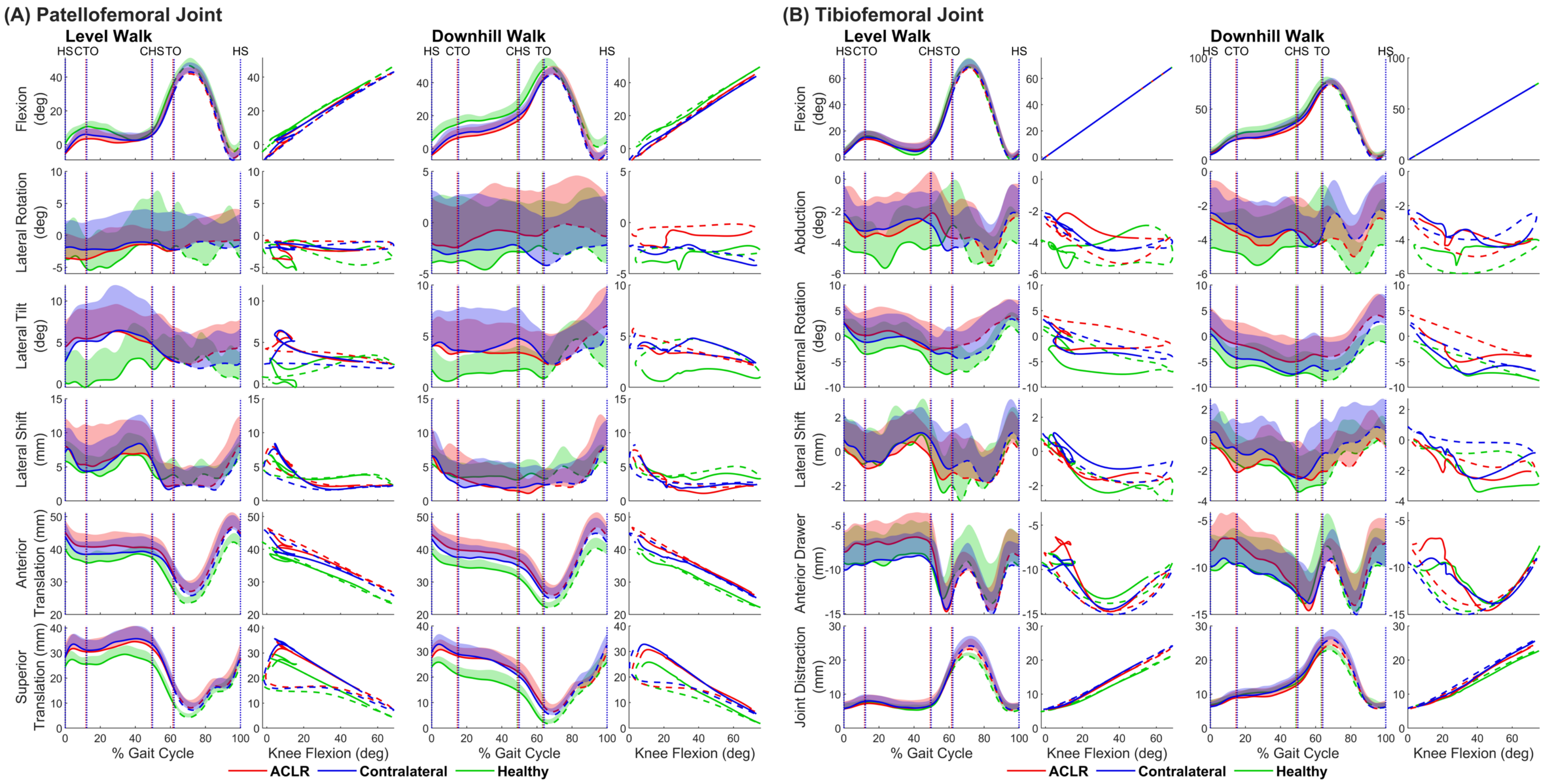

Supplementary Figure S3. Six-degree-of-freedom patellofemoral (panel A) and tibiofemoral (panel B) joint kinematics describing the rotations (rows 1-3) and translations (rows 4-6) of the patella and tibia with respect to the femur during level walking and downhill walking for the ACLR knee (red), contralateral knee (blue), and healthy knee (green). Kinematic parameters shown here were defined using the **joint coordinate system defined by Grood and Suntay (1983)**. Columns 1, 3, 5, and 7 show the mean and standard deviation calculated across all participants in each group plotted over the entire gait cycle, while columns 2, 4, 6, and 8 show the mean plotted against the knee (tibiofemoral) flexion angle. The solid and dashed lines represent the mean during the stance and swing phases of the gait cycle, respectively. The shaded areas represent one standard deviation from the mean. The vertical dotted lines indicate gait events: HS, heel-strike; CTO, contralateral toe-off; CHS, contralateral heel-strike; and TO, toe-off.

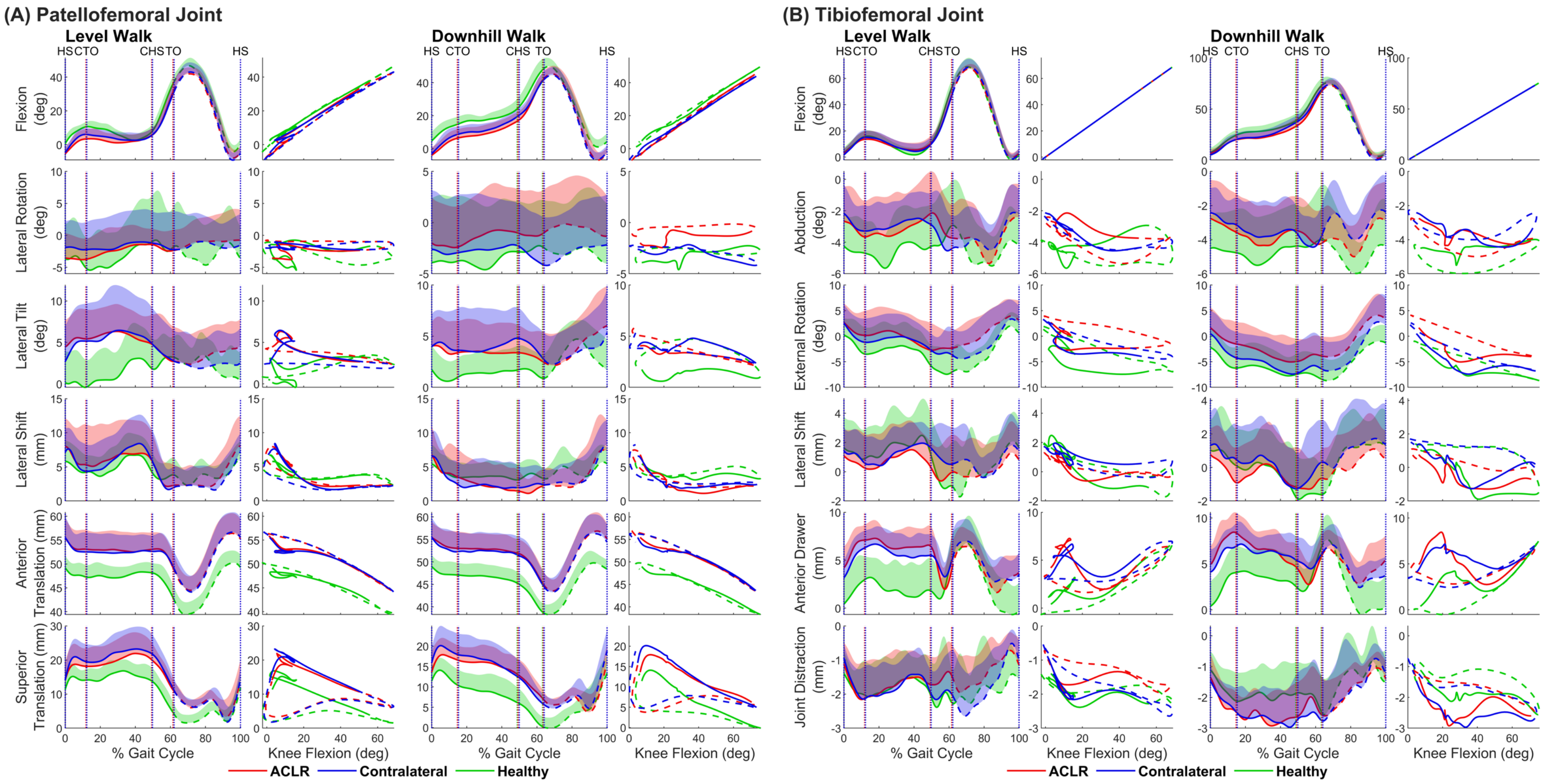

Supplementary Figure S4. Six-degree-of-freedom patellofemoral (panel A) and tibiofemoral (panel B) joint kinematics describing the rotations (rows 1-3) and translations (rows 4-6) of the patella and tibia with respect to the femur during level walking and downhill walking for the ACLR (red), contralateral (blue), and healthy (green) knees. Kinematic parameters shown here are identical to those in Figure 2 in the main paper and were defined using the **joint coordinate system defined by Gray *et al.* (2019)**. Columns 1, 3, 5, 7 show the mean and standard deviation calculated across all participants in each knee group plotted over the entire gait cycle, while columns 2, 4, 6, 8 show the mean plotted against the knee (tibiofemoral) flexion angle. The solid and dashed lines represent the mean during the stance and swing phases of the gait cycle, respectively. The shaded areas represent one standard deviation from the mean. The vertical dotted lines indicate gait events: HS, heel-strike; CTO, contralateral toe-off; CHS, contralateral heel-strike; and TO, toe-off.

S5. Ratios calculated to identify patella alta

Supplementary Table S3. Insall-Salvati ratio, modified Insall-Salvati ratio, and Blackburne-Peel ratio calculated for both knees of each ACLR participant and the tested (right) knee of each healthy participant.

ACLR/Healthy Participant	Insall-Salvati Ratio			Modified Insall-Salvati Ratio			Blackburne-Peel Ratio		
	ACLR	Contra.	Healthy	ACLR	Contra.	Healthy	ACLR	Contra.	Healthy
Participant #1	1.03	1.01	0.93	1.64	1.72	1.58	0.89	0.996	0.90
Participant #2	1.16	1.13	1.01	1.65	1.60	1.996	0.79	0.71	**1.13***
Participant #3	**1.31***	**1.40***	1.00	1.92	1.94	1.60	0.94	0.93	0.81
Participant #4	**1.38***	**1.39***	1.04	**2.11***	1.97	1.80	**1.14***	**1.06***	0.99
Participant #5	**1.28***	1.17	1.01	**2.05***	1.93	1.63	0.85	0.80	0.69
Participant #6	1.10	0.99	1.07	1.62	1.58	1.76	0.76	0.75	0.84
Participant #7	**1.50***	**1.38***	1.03	**2.20***	**2.09***	1.68	**1.03***	0.99	0.84
Participant #8	1.11	**1.22***	0.99	1.82	1.82	1.77	0.95	0.93	0.74
Participant #9	**1.32***	**1.35***	1.15	1.72	1.90	**2.05***	0.75	0.89	0.98
Participant #10	1.16	1.06	1.05	1.89	1.70	1.76	0.93	0.84	0.90
Participant #11	1.17	1.17		1.92	1.86		0.89	**1.02***	
Participant #12	**1.21***	1.15		1.78	1.68		0.94	0.82	
Participant #13	**1.29***	**1.36***		1.88	**2.04***		0.75	0.86	
Participant #14	1.16	**1.22***		1.84	1.94		0.86	**1.04***	
Participant #15	1.17	1.15		1.81	**2.21***		**1.07***	**1.20***	

*Bold type indicates patella alta identified with Insall-Salvati ratio ≥ 1.2, modified Insall-Salvati Ratio ≥ 2.0, or Blackburne-Peel ratio ≥ 1.0.
Abbreviation: Contra., contralateral.

References

Gray HA, Guan S, Thomeer LT, Schache AG, de Steiger R, Pandy MG. Three-dimensional motion of the knee-joint complex during normal walking revealed by mobile biplane X-ray imaging. *J Orthop Res*. 2019;37(3):615-630.

Grood ES, Suntay WJ. A joint coordinate system for the clinical description of three-dimensional motions: Application to the knee. *J Biomech Eng*. 1983;105(2):136-144.

Pieper S, Lorensen B, Schroeder W, Kikinis R. The NA-MIC Kit: ITK, VTK, pipelines, grids and 3D slicer as an open platform for the medical image computing community. In: *3rd IEEE International Symposium on Biomedical Imaging: Nano to Macro, 2006*. 2006:698-701.

Thomeer LT, Guan S, Gray HA, Pandy MG. Articular contact motion at the knee during daily activities. *J Orthop Res*. 2022;40(8):1756-1769.
